# Fair Allocation at COVID-19 Mass Vaccination Sites

**DOI:** 10.1001/jamahealthforum.2021.0464

**Published:** 2021-04-01

**Authors:** William F. Parker, Govind Persad, Monica E. Peek

**Affiliations:** Section of Pulmonary and Critical Care Medicine, University of Chicago, Chicago, Illinois; Sturm College of Law, University of Denver, Denver, Colorado; Section of General Internal Medicine, University of Chicago, Chicago, Illinois.

On February 26, 2021, the Federal Emergency and Management Agency (FEMA) announced 18 community vaccination centers in major cities capable of administering up to 6000 vaccines daily.^1^ Mass vaccination sites like these arrive amid staggering socioeconomic and racial disparities in COVID-19 vaccination. Black and Hispanic people are being vaccinated at less than half the rate of White people,^2^ despite being twice as likely to die of COVID-19.^3^ The wealth gap is similarly substantial, reaching up to a 65% difference between the wealthiest and poorest counties in Connecticut.^4^ The federal government is supporting mass vaccination sites, in part, to alleviate disparities, asserting that if sites are placed in dense urban areas then “equity is embedded”^1^ in their design.

While important, increasing supply and locating vaccines near underserved communities will not necessarily produce vaccine equity. Instead, if equity is not actively incorporated in eligibility criteria and sign-up processes, mass vaccination sites could siphon supply from disadvantaged communities and widen disparities. Developing a proactive plan to mitigate disparities is not only equitable but will maximize benefit by addressing disparate risks in underserved communities.

We propose 4 equity-advancing operational improvements to eligibility and sign-up processes at mass vaccination sites: (1) preregistration using existing information, (2) eligibility rules that recognize the greater burden of COVID-19 in underserved neighborhoods, (3) appointment assignment that prioritizes those with disadvantage, and (4) socioculturally informed outreach to lottery selectees.

## Preregistration Using Existing Information

Some mass vaccination sites are using first-come, first-served online systems that favor those with faster, reliable internet access and greater technological comfort, thus exacerbating inequity and negatively associating vaccine distribution with COVID-19 risk. A better approach is preregistration, which involves constructing a list of eligible patients using existing health system and government data, supplemented with alternative access pathways (including telephone, text message, and in-person registration) for individuals whom traditional data sets may miss, such as unhoused persons. Patients are selected from the preregistry with an equitable assignment mechanism and notified when it is time for their appointment by active outreach. This approach has already been embraced by the United Kingdom and some US jurisdictions. It should become the default at all FEMA sites for the remainder of the vaccination campaign.

## Align Eligibility Policies With Geographic Differences in COVID-19 Burden

Place is among the most powerful risk factors for acquiring and dying from COVID-19. One approach is to offer vaccines to all adults in severely impacted areas. Alternatively, eligibility criteria, such as age-based exclusions, could be adjusted to reflect community-level life expectancy. In the COVID-19 pandemic, the median age of death in minority communities is approximately 10 years earlier,^3^ likely driven by structural racism and place-based risk. Some states have already lowered age cutoffs for other groups, such as teachers and those with medical risk. Using zip codes to adjust age cutoffs in response to place-based risk would be an easily implementable improvement in equity and better than one-size-fits-all cutoffs.^5^

## Prioritize People Living in Disadvantaged and Undervaccinated Zip Codes

One approach to prioritizing neighborhoods with more risk and lower vaccination rates is a weighted lottery, which could be implemented using publicly available data. For example, in Chicago,^6^ the cumulative COVID-19 death rate to date ranges from 27 per 100 000 residents in zip code 60607, a predominantly high-income zip code with primarily White residents, to 324 per 100 000 residents in zip code 60649, which has a majority of Black and working-class residents. A weighted lottery assigns individuals a number of lottery tickets based on COVID-19 burden, such as the relative risk of death. For example, if the relative risk of COVID-19 mortality is 10.7 in a given zip code, those residents would each receive 11 tickets per draw. The lottery weights would need to be customized for each jurisdiction and could incorporate case positivity rates, hospitalizations, or community vulnerability indices.^7^

## Conduct Active Sociocultural Outreach to the Community

Following a census or canvassing model, initial invitations for vaccination should be delivered by simultaneous text message, telephone call, and email. Outreach workers should follow up, door-to-door if needed, with those who do not schedule appointments. Community-based advertising and education campaigns that leverage racial/ethnic minority media, community-based organizations and faith leaders, and other resources are needed. Community health workers and other trusted voices navigating patients to the point of vaccination will be critical. Mobile teams should be deployed from mass vaccination sites to vaccinate people facing transportation barriers.

## Rebutting Arguments for the First-Come, First-Served Model

First-come, first-served systems prioritize highly motivated vaccine recipients, potentially reducing no-show rates. This is attractive to officials who face tremendous pressure to vaccinate constituents rapidly and fear tension between speed and equity. While speed is important to maximize benefit, other dimensions are at least as important. Improving uptake in disadvantaged communities is likely to save more lives and mitigate COVID-19 spread because their residents are at higher risk of infection and poor outcomes. More importantly, automatic preregistration and weighted lottery assignments with active outreach will plausibly improve speed compared with passive first-come, first-served approaches, while enabling the prioritization of disadvantaged communities without onerous on-site verification.

State and local governments may fear the operational burden and costs of going beyond passive approaches. The experience at University of Chicago suggests that preregistration and a weighted lottery impose negligible costs. Zip code verification adds little additional burden beyond age verification. Thus, only active outreach and follow-up should require sustained operational costs, which forthcoming federal support for COVID-19 equity efforts can help meet. In the interim, building on existing community mobilization to support COVID-19 outreach efforts fosters the trust needed for successful mass vaccination campaigns.

Finally, some might argue that prioritizing disadvantaged communities is social engineering that unethically treats groups unequally. Fairness can be defined as to each an equal share, but in a pandemic, it is more appropriately defined as to each according to need. Every prominent vaccine allocation framework recognizes that fairness not only permits, but requires, treating people facing different risks and burdens differently. Our strategy should be used at least until hard-hit communities are well vaccinated.

## Equity Depends on Effective Implementation

Implementation will determine whether mass vaccination sites mitigate or worsen vaccine inequity. Operationally feasible approaches, including preregistration, weighted lotteries, and active outreach, can both prioritize disadvantaged communities and maximize population benefits. Mass vaccination sites should implement these strategies immediately.
